# On the Tests for Arsenic, in Reply to Mr. Hume

**Published:** 1818-09

**Authors:** John Prideaux


*For the London Medical and Physical Journal.*

### *On the Tests for Arsenic, in Reply to Mr. Hume;* by JOHN PRIDEAUX, Esq.

YOUR Journal for June has but just reached me, or I should sooner have noticed Mr. Hume's attention to my letter. I am glad to find he observed nothing new in it, and should have been still more so if he had favoured me with the result of his experience on the subjects of which it treated.—Of this, however, he gives me some hope at a future day. He says I put a forced construction on his language. It appears I have misunderstood his meaning; but it was the very confident tone of his language which induced me to intrude on your pages; and, if I have misconstrued it, I have many companions in error. But I did not mean to assert that Mr. Hume would "swear," &c. I only asked him the question, and his answer is satisfactory.

He seems to dispute the existence of phosphate of soda in the gastric fluid. I judged from analogy, and believe the opinion I gave is general; but I spoke too positively. It is, however, not very material as to the question in debate, because disease might as probably occasion the presence of phosphate of soda in the stomach as of phosphate of lime on the coats of the arteries, or of sugar from the kidnies. I cannot see that the manner in which I expressed my doubts on some points was unsuitable; nor should I imagine Mr. Hume's difficulty in replying to them, to have originated so much in my mode of speaking, as in the intricacy of the case. If the doubts were groundless, a very short answer might have cleared them up.

It is very probable that I have not duly estimated the use of ammoniaco-nitrate of silver, as I do not clearly see any advantage to be derived from its application. The cases Mr. Hume has cited seem to me in favour of this opinion. In the first, he does not employ ammoniaco-nitrate, but paper moistened with ammonia: in the second, he uses ammoniaco-nitrate, where ammonia only is necessary; because an excess of simple nitrate is always used when we throw down the muriatic acid, &c. When I said "ammoniaco-nitrate is not applicable," &c. I meant that it could not be used in that case to distinguish arsenic from phosphate of soda, and I thought it not easy to be misunderstood.

I think a reference to my last letter will show, that the remark, on my assertion of the greater certainty in applying the alkali first, is not suited to the case.

I should have been pleased if Mr. Hume had repeated what he states having so often mentioned, about the use of lime for getting rid of the phosphates. If he is guided only by theoretical views, he may, perhaps, have extended too the doctrine of incompatible salts. This doctrine, though very useful in manufacturing chemistry, has not sufficient precision for cases of this kind. Very few salts can be said to be absolutely incompatible, and almost all are so in some degree. I will not digress by giving examples, but confine myself to the case in point. If we let fall into a small test-tube four or five drops of a saturated solution of phosphate of soda, and nearly fill the tube with water; and, if we then dip the end of a fine glass tube into a weak solution of lunar caustic, and apply it to the surface of this fluid,—a precipitate of a pale-yellow colour will appear. If we prepare another tube with phosphate of soda in the same manner, and drop in some solution of pure nitrate of lime, a precipitate appears, and will subside in a few hours. If, after we have thrown down all that can be precipitated in this manner, we pour off the clear liquor into another tube, and apply the lunar caustic as before, we shall again have a precipitate, not so abundant and striking, but still decidedly yellow. In short, nitrate of silver is, as might be expected, a more delicate precipitant of phosphoric acid than nitrate of lime; and the latter cannot be depended on to remove this acid in our search for arsenic.

I have not tried the experiment which would be necessary to say whether, "supposing a little phosphate of soda, muriate of soda, and white arsenic, were mixed and exposed in a glass vessel to the heat of a spirit lamp, the arsenic would leave the salts, and attach itself to the upper part of the vessel." But I should imagine it would rise in the state of butter of arsenic, provided the ingredients were very intimately mixed; so as by solution in water and evaporation to dryness. But I do not clearly see how such a mixture should occur free from other ingredients.

I can by no means agree in the opinion that the sublimation of arsenic, in the state of white oxide, is the most certain method of collecting it for purposes of evidence. Though it may, perhaps, occupy a larger space, yet it is not so visible as the metal; and there are other substances which rise at moderate temperatures, and which, in small quantities, may bear some resemblance to the oxide; whilst I apprehend we are subject to no uncertainty of that kind with regard to the metal; nor do I see that it would be any proof of ignorance in a chemist, who should, in a case of life or death, reduce a quarter of a grain of arsenic to the metallic state, instead of precipitating it *viâ humidâ*. On the contrary, I think the certainty much greater, and the difficulty not worth considering.

I stated, in my former letter, and I thought with sufficient clearness, the reason why I considered chemical testimony liable to objections which did not apply to other technical evidence. I said, "courts of justice were not qualified to appreciate the sufficiency and abilities of the chemist." Other arts (here I must use that term) are followed for purposes of gain, and diplomas, certificates, and indentures of apprenticeship, can be produced in proof of the competency of the witness. But the philosophical chemist is differently circumstanced. His talents can be estimated only by those who understand the science, and there is no authority, except common fame, to which the court can appeal for the proof of his sufficiency.

The mineralogical anecdote, without the pointed reflexions it suggests, may seem a little misplaced; but I suppose there may be good reasons for its insertion.

It is not my business to defend the experiments used on the Launceston trial; yet I was present at that trial, and cannot but think they were properly in point. If I understood right, they were intended to show, that the effects produced on the tests, as the manner of using them was *detailed* on the part of the prosecution, might have been produced by other substances. The experiments were exactly repeated, on the part of the defence, on substances which might have been supposed to exist in the stomach, and appearances were produced similar to those which were adduced to prove the presence of arsenic. Mr. Hume's query, how could *decoction of onions* remain after so many evacuations? is at least as applicable to *arsenic:* and the extreme minuteness of the quantity was an important feature on the evidence, as must be observed by any who will read the published account of the trial.

I think, gentlemen, I have now replied to all the parts of Mr. Hume's letter that required my attention: perhaps I may have said more than was necessary. I should, indeed, have waited for the future communication he has given us to expect, but that I wished for further information with regard to the use of lime for removing phosphoric acid, and thought the query respecting the sublimation of oxide of arsenic from the mixture of salts, &c. ought not to be passed by without notice. And, whilst I have room on my paper, I may add, that the green precipitate from copper cannot be obtained from the fluid of the stomach to which arsenic shall have been added, unless the quantity of the poison be considerable. The copper only strikes a deep blue, which may be attributed to the presence of some salt of ammonia, probably the muriate, decomposed by the subcarbonate of potass and sulphate of copper. This may not always happen, but it has occurred in every instance where I have had opportunity of trying the experiment.

*Plymouth; July* 11, 1818.

